# A Collaboratively-Derived Science-Policy Research Agenda

**DOI:** 10.1371/journal.pone.0031824

**Published:** 2012-03-09

**Authors:** William J. Sutherland, Laura Bellingan, Jim R. Bellingham, Jason J. Blackstock, Robert M. Bloomfield, Michael Bravo, Victoria M. Cadman, David D. Cleevely, Andy Clements, Anthony S. Cohen, David R. Cope, Arthur A. Daemmrich, Cristina Devecchi, Laura Diaz Anadon, Simon Denegri, Robert Doubleday, Nicholas R. Dusic, Robert J. Evans, Wai Y. Feng, H. Charles J. Godfray, Paul Harris, Sue E. Hartley, Alison J. Hester, John Holmes, Alan Hughes, Mike Hulme, Colin Irwin, Richard C. Jennings, Gary S. Kass, Peter Littlejohns, Theresa M. Marteau, Glenn McKee, Erik P. Millstone, William J. Nuttall, Susan Owens, Miles M. Parker, Sarah Pearson, Judith Petts, Richard Ploszek, Andrew S. Pullin, Graeme Reid, Keith S. Richards, John G. Robinson, Louise Shaxson, Leonor Sierra, Beck G. Smith, David J. Spiegelhalter, Jack Stilgoe, Andy Stirling, Christopher P. Tyler, David E. Winickoff, Ron L. Zimmern

**Affiliations:** 1 Conservation Science Group, Department of Zoology, University of Cambridge, Cambridge, United Kingdom; 2 Society of Biology, Charles Darwin House, London, United Kingdom; 3 School of the Physical Sciences, University of Cambridge, Cambridge, United Kingdom; 4 Centre for International Governance Innovation (CIGI), Waterloo, Ontario, Canada; 5 International Institute for Applied Systems Analysis (IIASA), Laxenburg, Austria; 6 Natural History Museum, London, United Kingdom; 7 Department of Geography, University of Cambridge, Cambridge, United Kingdom; 8 Ecological Continuity Trust, Charles Darwin House, London, United Kingdom; 9 Centre for Science and Policy, University of Cambridge, Cambridge, United Kingdom; 10 British Trust for Ornithology, The Nunnery, Thetford, United Kingdom; 11 Department of Environment, Earth and Ecosystems, The Open University, Milton Keynes, United Kingdom; 12 Parliamentary Office of Science and Technology, Houses of Parliament, London, United Kingdom; 13 Harvard Business School, Boston, Massachusetts, United States of America; 14 School of Education, University of Northampton, Park Campus, Northampton, United Kingdom; 15 Belfer Center for Science and International Affairs, Harvard Kennedy School of Government, Cambridge, Massachusetts, United States of America; 16 Ovarian Cancer Action, London, United Kingdom; 17 Pfizer Ltd, Kent, United Kingdom; 18 Centre for the Study of Knowledge, Expertise and Science, Cardiff University, School of Social Sciences, Cardiff, United Kingdom; 19 Faculty of Education, University of Cambridge, Cambridge, United Kingdom; 20 Department of Zoology & Oxford Martin School Institute of Biodiversity, University of Oxford, Oxford, United Kingdom; 21 Australian National Institute for Public Policy, The Australian National University, Canberra, Australian Capital Territory, Australia; 22 York Environmental Sustainability Institute, University of York, York, United Kingdom; 23 The James Hutton Institute, Aberdeen, United Kingdom; 24 Department of Earth Sciences, University of Oxford, Oxford, United Kingdom; 25 Centre for Business Research, Judge Business School, University of Cambridge, Trumpington Street, Cambridge, United Kingdom; 26 Science, Society and Sustainability (3S) Group, School of Environmental Sciences, University of East Anglia, Norwich, United Kingdom; 27 Defence Science & Technology Laboratory, Fareham, United Kingdom; 28 Department of History and Philosophy of Science, University of Cambridge, Cambridge, United Kingdom; 29 Evidence and Analysis Team, Natural England, London, United Kingdom; 30 The National Institute for Health and Clinical Excellence, MidCity Place, London, United Kingdom; 31 Behaviour and Health Research Unit, University of Cambridge, Institute of Public Health, Forvie Site, Cambridge, United Kingdom; 32 Department of Chamber and Committee Services, House of Commons, London, United Kingdom; 33 SPRU - Science and Technology Policy Research, Freeman Centre, University of Sussex, Brighton, United Kingdom; 34 Judge Business School, University of Cambridge, Cambridge, United Kingdom; 35 Department for Environment Food and Rural Affairs, Strategic Evidence, London, United Kingdom; 36 College of Business and Economics, The Australian National University, Canberra, Australian Capital Territory, Australia; 37 Faculty of Social and Human Sciences, University of Southampton, Southampton, United Kingdom; 38 Royal Academy of Engineering, London, United Kingdom; 39 Centre for Evidence-Based Conservation, School of Environment, Natural Resources and Geography, Bangor University, Bangor, Gwynedd, United Kingdom; 40 Department for Business Innovation and Skills, London, United Kingdom; 41 Wildlife Conservation Society, Bronx, New York, United States of America; 42 Delta Partnership, Central Hall Westminster, London, United Kingdom; 43 Sense About Science, London, United Kingdom; 44 Biochemical Society, Charles Darwin House, London, United Kingdom; 45 Statistical Laboratory, Centre for Mathematical Sciences, University of Cambridge, Cambridge, United Kingdom; 46 Science Policy Centre, The Royal Society, London, United Kingdom; 47 Department of Environmental Science, Policy & Management, University of California, Berkeley, California, United States of America; 48 PHG Foundation, Cambridge, United Kingdom; IUMSP, University Hospital Lausanne, Switzerland

## Abstract

The need for policy makers to understand science and for scientists to understand policy processes is widely recognised. However, the science-policy relationship is sometimes difficult and occasionally dysfunctional; it is also increasingly visible, because it must deal with contentious issues, or itself becomes a matter of public controversy, or both. We suggest that identifying key unanswered questions on the relationship between science and policy will catalyse and focus research in this field. To identify these questions, a collaborative procedure was employed with 52 participants selected to cover a wide range of experience in both science and policy, including people from government, non-governmental organisations, academia and industry. These participants consulted with colleagues and submitted 239 questions. An initial round of voting was followed by a workshop in which 40 of the most important questions were identified by further discussion and voting. The resulting list includes questions about the effectiveness of science-based decision-making structures; the nature and legitimacy of expertise; the consequences of changes such as increasing transparency; choices among different sources of evidence; the implications of new means of characterising and representing uncertainties; and ways in which policy and political processes affect what counts as authoritative evidence. We expect this exercise to identify important theoretical questions and to help improve the mutual understanding and effectiveness of those working at the interface of science and policy.

## Introduction

The importance of understanding and using science for public policy-making has long been recognised [1], but recent years have seen a growing debate over how this is best achieved [2]–[4]. Still more recently, ‘evidence-based policy’ has become the desired norm in many fields (even if its meaning is still disputed), and this has led to a greater embedding of scientists, both natural and social, alongside other specialists in public policy–making processes. In many governments, scientists are engaged at a senior level. The US, for example, has the President's Council of Advisors on Science and Technology, while the UK has Chief Scientific Adviser posts in all government departments, in addition to a Government Chief Scientific Adviser with a place in some Cabinet Committees.

In spite of their acknowledged importance however, relations between science and policy are sometimes troubled [5], and periodically erupt into controversy. Prominent examples include the acrimonious debate over scientific understandings of climate change [6], further inflamed by the ‘Climategate’ email controversy, disputes over the use of genetically modified crops and foods in Europe, the failure to acknowledge the risk of possible BSE transmission to humans [7], and conflict over stem cell research, which is particularly acute in the United States. In 2009, the public sacking of the Chair of the UK Advisory Council on the Misuse of Drugs began a row not only about appropriate policy (in this case for drugs classification), but also about the proper place of independent scientific advice in the policy-making process. Such troubles are symptomatic of the complexity of science-policy interactions, and suggest that there is still much to understand about the nature of scientific authority and processes of policy formation and change [8]–[10].

Against this backdrop, this paper reports the results of an exercise that sought to identify the most important outstanding questions in this domain. Precedents for attempts to identify ‘key questions’ go back to the learned civic societies of enlightenment England and France. For example, the Royal Society for the Encouragement of Arts, Manufactures, and Commerce (founded 1752) and the French National Institute (1795–1983) identified specific policy-relevant questions for which they offered prizes to promote commercial and social applications of science [11]. Other examples include Hilbert's famous set of mathematical questions [12], Paul Erdös' posing of mathematical questions with cash prizes for those who solved them [13] and Steffen *et al's* [14] listing of questions in the environmental sciences. Contemporary ‘top down’ examples include the US National Research Council, in its assessment of strategic directions for the geographical sciences [15], and the International Council for Science, with its Grand Challenges in Global Sustainability Research [16].

We have adopted a rather different, bottom-up, approach, bringing together researchers, policy makers and practitioners with interests in relations between science and policy to identify priority, researchable questions in this field. The method is similar to that used in conservation biology [17]–[21] and agricultural science [22]. Previous exercises have been remarkably influential [23]. For example, two of the resulting papers [17], [22] were the most downloaded ever from their respective journals, and one [17] was explicitly cited as the basis for the priority research questions identified within the UK Marine Science Strategy [24]. Our aim has been to identify key questions which, if addressed through focused research and enquiry, might not only help resolve important theoretical challenges but might also improve the mutual understanding and effectiveness of those who work at the interface of science and policy.

The questions presented below were generated through a democratic, transparent and collaborative process similar to those used in previous exercises [23]. There are interesting differences in this case, however, because the existence of a pre-determined research and policy community is much less evident. Participants were therefore selected to cover a wide range of academic disciplines (including the biological, environmental, medical, physical, and social sciences) as well as governmental and non-governmental organisations, consultancies and industry. Initially, each participant was invited to produce a list of questions, consulting widely if they wished to do so (see the Materials and Methods section below). The 239 questions submitted at this first stage are presented in the Material S1. A process of voting, deliberation and further voting (the final stages of which took place at a meeting of participants over two days) subsequently reduced the initial list to a final set of 40 questions. During this process the questions were also redrafted and grouped thematically. They are presented in the following section, ordered by theme but not in rank order.

The outcomes of an exercise such as this are inevitably influenced by the composition of the set of participants, as well as by the process. Clearly, therefore, the results are not ‘reproducible’ (in the sense that a re-run with different people could be expected to produce exactly the same set of questions). Nevertheless, if the exercise were to involve a similarly large and diverse group of participants, and were to be conducted, like this one, through several rounds of voting, deliberation and editing, we consider it highly likely that broadly similar general themes would emerge. This is, of course, an empirically testable proposition.

## Results

### Understanding the role of scientific evidence in policymaking

1. How do different political cultures and institutions affect the acquisition and treatment of scientific evidence in policy formulation, implementation and evaluation?
2. How do scientists and policy makers recognise and convey the limitations of scientific advice?
3. At what stages during the development of policy does scientific evidence have the greatest impact on the decisions made?
4. Under what conditions does scientific evidence legitimise political decisions?
5. What roles have science and other forms of expertise played in international governance regimes, such as the World Trade Organisation?
6. Are there conditions under which scientific evidence may help resolve value-laden conflict and if so, what are those conditions?
7. What factors affect the utility and legitimacy of formal decision support, assessment and evaluation tools, and their adoption (or otherwise) by policy makers?
8. What influences the form and application of monitoring and evaluation practices in the development of policy informed by science?

### Framing questions, sourcing evidence and advice, shaping research

1. How do policy makers decide which questions they should ask their expert advisors and when in the policy cycle they should be asked?
2. What are the most effective mechanisms for identifying the evidence required to inform policy-making on new and emerging problems?
3. How, and with what consequences, have the sources of scientific evidence and advice used by policy makers changed over recent decades?
4. In what ways do different political cultures shape the frameworks through which evidence and advice are sourced?
5. In what circumstances are policy problems likely to require the inclusion of experts with conflicting views?
6. When is it considered appropriate to consult experts with conflicting views, and what mechanisms can ensure that this takes place?
7. What factors influence whether different disciplines are included effectively when defining and addressing complex policy problems?
8. What are the mechanisms by which budgetary pressures and societal constraints on policy-making influence the prioritisation and funding of research?
9. What is the effectiveness of different techniques for anticipating future policy issues requiring science input?

### Advisory systems and networks

1. How are national science advisory systems constructed and to what extent do different systems result in different outcomes?
2. How and why does the role of scientific advice in policy-making differ among local, regional, national and international levels of governance?
3. Which commissioning and operational arrangements lead to the most effective use of science in policy-making?
4. Policy makers typically use networks of experts, formal and informal. How does the structure and composition of such networks influence the outcomes of decision making?
5. How do different ways of using and organising in-house scientific expertise affect the quality and use of scientific evidence and advice in policy-making?
6. What are the consequences of different approaches to institutionalising, professionalising and building capacity in the exchange of knowledge between science and policy?
7. How can the effectiveness of knowledge-brokering [5] be assessed?

### Policy making under conditions of uncertainty and disagreement

1. How is agreement reached on what counts as sufficient evidence to inform particular policy decisions?
2. How is scientific evidence incorporated into representations of, and decision-making about, so-called “wicked” problems, which lack clear definition and cannot be solved definitively?
3. Can distinctions be made in scientific advice between facts and values; to the extent that this is possible, how effective are policy makers in distinguishing them and what factors influence their effectiveness?
4. How can risks, and the associated uncertainties, complexities, ambiguities and ignorance, be effectively characterised and communicated?
5. How do policy makers understand and respond to scientific uncertainties and expert disagreements?
6. Do different approaches to building consensus, or illuminating lack of consensus, result in different consequences for policy and, if so, why?

### Democratic governance of scientific advice

1. What factors (for example, openness, accountability, credibility) influence the degree to which the public accept as trustworthy an expert providing advice?
2. What governance processes and enabling conditions are needed to ensure that policymaking is scientifically credible, while addressing a perceived societal preference for policy processes that are more democratic than technocratic?
3. How might the attitudes and values of diverse publics relating to science and technology, and their governance, be incorporated effectively into debates about the use of evidence in policy-making?
4. What has been the influence of scrutinising institutions, such as those of legislative bodies (e.g. Parliament, Congress, National Assembly or Bundestag) on the roles of science in policy-making?
5. What are the implications for their effectiveness of opening up expert advisory processes to different forms of transparency?
6. What are the implications for science-policy relations, and for the democratisation of science, of novel methods of engagement and dissemination (such as citizen science, and new media technologies, including social media)?

### How do scientists and policy makers understand expert advisory processes?

1. What factors shape the ways in which scientific advisors and policy makers make sense of their own and each other's roles in the policy process?
2. How and why have the conceptual models of science-policy relations held by policy makers, scientists and other stakeholders changed over time, and with what consequences?
3. How is guidance on the handling and communication of risk, uncertainty and ambiguity interpreted by policy makers, and what impact do their views have on the uptake and implementation of recommendations?
4. What impact has research on the relationship between science and policy actually had on science policy?

## Discussion

Although it may seem self-evident that policy should be informed by scientific understanding, and should therefore be evidence-based, this normative assumption is itself based on surprisingly weak evidence. Debates continue, for example, about what exactly constitutes good evidence, where and how such evidence should be sought, and at what stage in the policy process different forms of evidence might be more or less appropriate. That such debate persists reflects the fact that there are many open questions about the nature of science-policy interactions, as this exercise has revealed. In short therefore, we need to ask not just how science can best inform policy, but also how policy and political processes affect what counts as authoritative evidence in the first place.

Jasanoff's [2] seminal study of science advisers showed that the value of science in policy stemmed in part from its capacity for detailed engagement with practical policy problems. At the same time, the authority of science was seen to depend on maintaining its independence from politics through separation, in what has been referred to as ‘boundary-work’ [25]–[26]. Rhetorical commitments in the policy world to a clear distinction between facts and values were ever-present. Since then, however, experience in many different contexts, both national and issue-based, has brought about a much greater awareness of the processes of interconnection among science, politics, policy-making and publics [27]–[28]. As Bijker *et al.* [8] note, an appreciation of the limits of science as an impartial arbiter among policy options comes at exactly the moment when demands for scientific input to policy are increasing. This tension is reflected and articulated in many of the questions generated by the interdisciplinary exercise reported here.

The six broad themes around which the questions have been organized constitute a potential framework for formulating research priorities, if we seek to develop better understandings of how science-policy interactions occur, and of evidence-based policy in practice. Beginning with a set of questions that consider the formal role that science might be expected to play in policy-making, we move on to two sets of more empirical questions about the ways in which science is selected and evaluated within the policy process, and how advisory processes actually work as an established system of governance; both sets of questions bear on the issues of expertise and authority. The following two themes then consider some of the limits to scientific knowledge, specifically in relation to inherent uncertainty and pervasive interdisciplinarity, and the roles of democratic participation and accountability in science-policy interactions. Taken together, these first five themes suggest a maturing appreciation of complexity and mutual interdependence in these relations; of the value and ubiquity of science in contemporary policy making; of the limits of ‘speaking truth to power’; and of the considerable effort that goes into the routine tasks of managing science policy.

Perhaps most interestingly, the final theme opens up a series of questions about how reflection on, and better understanding of, the nature of science-policy relations might help to improving the ways in which scientific evidence and advice is commissioned, constructed and transmitted when developing forms of evidence-based policy. The exercise reported here may therefore be seen as a contribution to developing a broad research agenda for investigating this critical, complex and contested relationship, perhaps in ways that could enhance its capacity to bring the best available knowledge effectively to bear on twenty-first century problems.

## Materials and Methods

The methods used in this exercise are similar to those described in Sutherland *et al*. [23] based on the experience of a series of attempts to identify priority questions [17]–[19], [21]–[22], [29]. The 52 participants were selected to cover a wide range of approaches to science and policy across government, non-governmental organisations, academia and industry. All participants are authors of this paper; the address list indicates their affiliations.

Each participant was permitted to consult widely among their own colleagues in obtaining an initial list of questions. We asked participants how many people they had actively consulted (for example, in workshops, meetings or email discussions, but not including those who were sent details and did not respond). From the responses we know at least an additional 83 beyond the participants were involved in devising questions. In total, 239 questions were submitted. These questions were collated into twelve themes. They were then sent to all participants, who were asked to select around fifty that they considered to be the most important. 29 voted. 11 questions obtained no votes. Participants were also invited to suggest alternative wording.

The final screening took place at a two-day workshop held in Cambridge in April 2011. On the first evening the process was discussed and potential misunderstandings and problems resolved. Prior to the meeting all participants had been provided with the number of votes for each question and any suggested rephrasing. On the following day, the workshop was divided into three 105 minute sessions, each with four groups meeting in parallel – twelve discussion groups in total, one discussing each question theme. Each group was charged with reducing one of the twelve-question themes to three priority questions plus a ranked list of three reserves. A rapporteur (from outside the team) was assigned to each session to incorporate changes to questions and capture the shortlist of the emergent top six; participants observed the editing process (projected onto a screen) as it was being carried out.

Each group had a different, pre-allocated chair (three of whom had previous experience of chairing sessions in similar exercises). A guidance note for chairs suggested that early decisions could be made to drop questions with zero or very few votes from the initial voting round; and also that groups of questions that clearly addressed similar issues could be identified. This process was designed to assist the group in identifying priorities, removing redundancy, and rewording questions to eliminate overlap and improve clarity. The group then voted on the remaining questions in order to select those considered the most important. Chairs also needed to maintain structure and direction in what were invariably vigorous and challenging deliberations.

In a final plenary session chaired by WJS, the top 36 questions (three from each of the twelve groups) were presented as a printed list to each participant to identify overlaps, problem questions and potential clarifications. Editing was again projected onto a screen and so was visible to all. When disagreement could not be resolved by discussion, decisions about inclusion or exclusion of questions, and about specific wording, were made by majority voting. Seven questions were removed by this process. The 12 top-ranking second-level questions were examined and the top 6 of these selected by voting (each participant having 6 votes). They were then discussed further to resolve any overlaps. The next 12 secondary questions were examined along with the remaining top ranked questions and the final five questions selected with each participant having five votes.

Selected questions were then clustered into 6 categories by placing related questions together, and edited by the entire group to produce the questions set out in this paper. During this process, after discussion and another round of voting, one question was removed and one short-listed question was added. As with previous exercises [23] most questions changed considerably from initial submission to final product. Forty-three participants made comments on or edits to the 64 successive versions of the paper that were circulated to all participants.

We did not obtain ethics approval for this exercise, as it was agreed from the outset that all those participating in the voting and selection of questions were to become authors of the resulting paper. However, all submitted questions were treated anonymously; and an agreement was made to publish in an open-access journal, if possible, in order to facilitate general accessibility for those in policy communities.

## Supporting Information

Material S1 The questions submitted to this exercise.
(DOCX)

## References

[1] Rothschild Report (1971). *The organisation and management of government research and development.* (Cmnd. 4814).

[2] Jasanoff S (1994). The Fifth Branch: Science Advisers as Policymakers.

[3] Clark TW (2002). The Policy Process.

[4] Cohen S (2006). Understanding Environmental Policy.

[5] Pielke RA (2007). The Honest Broker.

[6] Hulme M (2009). Why We Disagree About Climate Change: Understanding Controversy, Inaction and Opportunity.

[7] Phillips NA (2000). The BSE inquiry.

[8] Bijker W, Bal R, Hendriks R (2009). The Paradox of Scientific Authority: The Role of Scientific Advice in Democracies..

[9] Owens S (2005). Making a difference: some reflections on environmental research and policy.. T I Brit Geogr.

[10] Owens S, Lentsch J, Weingart P (2011). Knowledge, advice and influence: the role of the UK Royal Commission on Environmental Pollution 1970–2009.. The Politics of Scientific Advice: Institutional Design for Quality Assurance.

[11] Staum MS (1982). Images of Paternal Power: Intellectuals and Social Change in the French National Institute.. Can J Hist.

[12] Hilbert D (1902). Mathematical problems.. B Am Math Soc.

[13] Hoffman P (1998). The Man who Loved only Numbers: The Story of Paul Erdös and the Search for Mathematical Truth.

[14] Steffen W, Sanderson A, Tyson PD, Jäger J, Matson PA (2004). Global Change and the Earth System: A Planet Under Pressure.

[15] Committee on Strategic Directions for the Geographical Sciences in the Next Decade, National Research Council of the National Academies (2010). Understanding the Changing Planet: Strategic Directions for the Geographical Sciences.

[16] ICSU (2010). Earth System Science for Global Sustainability: The Grand Challenges..

[17] Sutherland WJ, Armstrong-Brown S, Armsworth PR, Brereton T, Brickland J (2006). The identification of one hundred ecological questions of high policy relevance in the UK.. J Appl Ecol.

[18] Sutherland WJ, Adams WM, Aronson RB, Aveling R, Blackburn TM (2009). An assessment of the 100 questions of greatest importance to the conservation of global biological diversity.. Conserv Biol.

[19] Sutherland WJ, Albon SD, Allison H, Armstrong-Brown S, Bailey MJ (2010). The identification of priority opportunities for UK nature conservation policy.. J Appl Ecol.

[20] Morton SR, Hoegh-Guldberg O, Lindenmayer DB, Olson MH, Hughes L (2009). The big ecological questions inhibiting effective environmental management in Australia.. Austral Ecol.

[21] Fleishman E, Blockstein DE, Hall JA, Mascia MB, Rudd MA (2011). Top 40 priorities for science to inform conservation and management policy in the United States.. BioScience.

[22] Pretty JJ, Sutherland WJ, Ashby J, Auburn J, Baulcombe D (2010). The top 100 questions for global agriculture and food.. Int J Agric Sustain.

[23] Sutherland WJ, Fleishman E, Mascia MB, Pretty P, Rudd MA (2011). Methods for collaboratively identifying research priorities and emerging issues in science and policy.. Methods Ecol Evol.

[24] DEFRA (2010). UK marine science strategy: shaping, supporting, co-ordinating and enabling the delivery of world class marine science for the UK, 2010–2025..

[25] Gieryn TF (1983). Boundary-Work and the Demarcation of Science from Non-Science: Strains and Interests in Professional Ideologies of Scientists.. Am Soc Rev.

[26] Star SL, Griesemer JR (1989). Institutional Ecology, ‘Translations’ and Boundary Objects: Amateurs and Professionals in Berkeley's Museum of Vertebrate Zoology, 1907–39.. Soc Stud Sci.

[27] Nowotny H, Scott P, Gibbons M (2001). Re-thinking Science: Knowledge and the Public in an Age of Uncertainty.

[28] Oppenheimer M (2011). What Roles Can Scientists Play in Public Discourse?. Eos.

[29] Rudd MA, Beazley KF, Cooke SJ, Fleishman E, Lane DE (2011). Generation of Priority Research Questions to Inform Conservation Policy and Management at a National Level.. Conserv Biol.

