# Supplementary material for: A Collaboratively-Derived Science-Policy Research Agenda
Source: PLoS One. 2012 Mar 9;7(3):e31824. doi: 10.1371/journal.pone.0031824 (PMC3302883; doi:10.1371/journal.pone.0031824)
Supplement: Material S1 — The questions submitted to this exercise. (DOCX) [file pone.0031824.s001.docx]

**S1 Supplementary material The questions submitted to this exercise.**

1. Do differences across countries in the extent to which science informs policy make any difference in terms of successful policy impacts at country level?
2. Do differences across countries in the extent to which science informs policy make any difference in terms of successful policy impacts at individual industry or company level?
3. What forms of government advisory and governance structures enhance the use of scientific advice in policy making?
4. Which science communication methods (journal publications, policy briefs, workshops, targetted presentations, etc) are most successful at reaching policy makers?
5. What are the most common responses by policy makers to science communications in different forms (i.e. measure of success by ‘action’)?
6. How do you communicate best to potential advisors the science evidence needs of policy makers?
7. Are standing Science Advisory Councils or similar bodies the best ways of ensuring independent, audited advice to government and other bodies
8. To what degree should Chief Scientific Advisors be independent of government?
9. When should external advice on science policy be confidential?
10. Is the divide between evidence and policy real?
11. How do you deal with policy advice in areas where people have different non-evidence based values and ethics?
12. How do you break down silos in science and across disciplines to deliver better science?
13. How do established regulatory bodies operate when confronted with new scientific research that is disruptive to their existing methods of enforcement or determining facts?
14. Why is there so little harmonization, even among advanced industrialized countries, in fundamental understandings of risk related to consumer products?
15. What are the right metrics to measure the effectiveness of policy?
16. What politics underlie demands for cost-benefit analysis in policy-making?
17. How do we best measure benefits and costs of cost-benefit analyses?
18. What responses are appropriate and legitimate when organized non-state actors establish alternative forms of policy-relevant knowledge and contest existing policy?
19. What makes a country good at research?
20. What makes a university good at research?
21. To what extent do policy and evidence communities understand each other? What are their respective worldviews, framings, language, cultures, ways of working and motivations? What is done to help each understand the other and adjust their working to accommodate the differences and maximise the synergies?
22. What counts as ‘effective’ knowledge brokering? – what criteria would be used in practice to judge the quality of knowledge brokering and how could any evaluation enable learning to take place.
23. What effective means exist (or could be created) to recognise, incentivise and reward knowledge brokering as a professional skill in its own right.
24. How can we effectively avoid the Wittgensteinian trap between evidence (which is only ever a statement of partial knowledge about how the world was or maybe is) and policy (which is a statement of how the world should be)...what are the practical limits of evidence-based policy; set against evidence-informed or evidence-bounded policy...recognising the 5 Ps: evidence is ultimately Partial, Plural and Provisional evidence and policy is ultimately Political and Pragmatic.
25. In looking at a policy issue and its relationship with evidence, how might we assess: the degree of clarity regarding the policy issue; the degree of consensus around the meaning of the evidence; what counts as relevant and appropriate expertise’ under different variations of the above.
26. How can degrees of uncertainty be conveyed to policy makers and incorporated into policy?
27. Should policy makers draw a distinction between “hard” and social sciences and take greater account of the former than latter
28. Is social science an art rather than a science?
29. Is there a growing anti-science ideology in democracies? Why, and what effect will it have on policy?
30. Is peer review an adequate mechanism to check and validate science? What assurance can policy makers take from per review?
31. Is qualitative or quantitative evidence of greater use in formulating policy?
32. When a policy firmly based on the best science and robust evidence fails, who should carry the political responsibility?
33. What are the cognitive barriers separating the trained scientist and the non-scientist; how do the different mind sets affect the ability to communicate scientific understanding and concepts to non-scientists, and how does scientific training get in the way of understanding non-scientific and value aspects of lay questions to scientists?
34. What are the epistemic models of policy makers and how do they compare/contrast to those of scientists?
35. What are the barriers to communication between risk specialists and those without routine familiarity with concepts of risk, uncertainty and ambiguity?
36. What is the most effective way to communicate very large and very small quantities, outside normal everyday limits? The Rothschild paradigm: customers for applied science are best positioned to define their needs; intelligent customer capability derives from employing an in house Chief Scientist has ruled government research commission since 1975. Has it worked – i.e. is government a more intelligent user of science (how would we know) and if so, is it because of the Rothschild reforms?
37. Horizon scanning is obviously sensible to identify unforeseen problems – but what is the most effective approach to separating signal from noise at long time range?
38. Donald Rumsfeld identified three categories of issues for policy makers (known knowns, known unknowns and unknown unknowns) and focused on the last, which is the subject of horizon scanning. He missed the fourth, unknown knowns; what is the best way of ensuring you gain access to sources of knowledge that could help when you don’t know enough to ask a question, or even recognise that there might be a question to be asked?
39. What are the set of knowledge, skills and aptitudes that a policy maker needs to be able to understand scientists? A scientist needs to be able to advise policy makers? And how have these been demonstrated in case studies?
40. Why and when is evidence to inform policy considered 'sound'?
41. In exploring the connections between 'science' and 'policy', are boundaries between these categories more difficult to draw in some cases than in others, or are they always and inevitably negotiated?
42. Why is the linear-rational model of the science-policy relationship so tenacious?
43. In complex democracies grappling with complex issues, does a system emphasising evidence-based policy inevitably slide towards technocracy?
44. How has policy been defined in the literature and to what extent is it possible to establish an agreed understanding of how policy should be developed in the UK?
45. What are the quality dimensions by which policy can be assessed? And how should they be weighted?
46. Can there ever be such a thing as value-free policy? And if so, would it be desirable?
47. What processes might be developed to ensure that policy makers can explicitly distinguish between the scientific basis of and the values based inputs to policy development?
48. What is the relationship between fact and value in policy development as it exists at present? What should it be?
49. It is a truism that the public should be included in the policy making process. But exactly when, and for what purpose is their input desirable and useful, and in what circumstances might their views have a negative impact? Should their views be confined only to matters concerning values?
50. What role should citizens have in setting the agenda for or determining the priorities in policy development?
51. What specific part should academics (in their University role) play in determining policy?
52. How does this differ (if it does) from those who work in independent policy institutes?
53. How independent are ‘independent policy institutes’? Are they ever value free? Can they be and should they be?
54. Are there circumstances in which it can be justified to keep secret the evidence used in policy development from the public? If so, what are they?
55. What are the opportunities and risks associated with the use of novel ‘open’ models for the science-policy interface?
56. Are there successful models that enable policy makers and scientists to collaborate on large-scale, cross institutional and multidisciplinary challenge based research? If so, what are their keys to success and how is success measured and evaluated?
57. What are the root causes of cultural barriers between the research and public policy sectors, what frameworks can be used to describe them, and what action can be employed to enhance the relationships between them?
58. What strategies have been successful in aligning the policy development process with long term, breakthrough, transformational science? What have been the keys to success and how is success measured and evaluated?
59. When scientific evidence is unclear or uncertain, what methods of communication are best suited at the interface between science and policy?
60. When conflict exists between scientific evidence and other policy imperatives, what models for decision making and communication are most effective?
61. Globally, what models for successful interfaces and knowledge transfer mechanisms between disparate cultures exist, and how could these models be transferred to the science-policy interface?
62. What skills and cultures are required to transfer science outcomes to policy in the public, private and third sector, and what policy initiatives and relationships can be employed to identify, develop, support and grow these? What is needed to facilitate multi-directional movement of staff between these areas?
63. What is the distribution of political allegiance amongst scientists? Does this have an impact on the quality of the science-policy relationship and effectiveness of knowledge transfer?
64. What incentives exist for both policy makers and scientists to collaborate, and do they align to deliver excellent outcomes?
65. What is the potential for new visualisation technologies to improve the use of science in policy-making?
66. Would improved integration between the natural/physical sciences and the social sciences and humanities improve the use of science in policy-making?
67. How can newer academic disciplines be used to apply different perspectives to public sector issues?
68. How can the research sector more effectively contribute to the policy testing and evaluation areas?
69. How can disparate areas of academic discipline be better harnessed to collectively contribute to specific policy problems?
70. Can scientific evidence ever reign supreme in a political environment?
71. How does the English parliament compare with other legislatures in the resources made available to legislators to understand science issues? Could we do better?
72. What is the best way to present ‘scientific evidence’ to policy makers?
73. What influences policy makers in their decision making and where does ‘scientific evidence’ fit in this process?
74. What is the role of different influences on different groups of policy makers? i.e. what sort of science/evidence do local policy makers take into account when producing policy, does this differ between policy domains such as between social care, healthcare and education or the Arts, does this differ across professional groups for example between doctors and nurses?
75. How can you assess the uptake and impact of scientific evidence in policy?.
76. What could/should be/is the role of Multi-criteria decision analysis (MCDA) in public decision-making; specifically in health care and, in decisions around reimbursement of medicines (and medical devices/diagnostics/interventional procedures)?
77. What are the pros/cons or strengths/weaknesses of techniques used for the operationalisation of multi-criteria decision analysis (MCDA) in public decision-making?
78. There are many different MCDA methods used in practice today for public decision-making. However, very different results can be obtained for the same problem depending on the methods used. Therefore understanding the strengths/weaknesses of the different techniques would be essential
79. At what stages of policy development and decision-making is scientific evidence or advice sought?
80. How is scientific advice most effectively communicated to policy-makers?
81. Are different types of scientific communication most effective at different policy-development stages? Why?
82. Are there significant differences in what is most effective for different scientific disciplines and within different policy teams? Why?
83. How is science communicated within and between policy units
84. By what criteria are scientific data / evidence assessed?
85. How is apparently conflicting data reconciled and uncertainty or gaps in knowledge addressed?
86. How do policy-makers identify advisors and build networks. How widely do policy-makers actively seek to consult?
87. How are policy-makers networks balanced across different disciplines and across different institutions?
88. To what extent are scientists aware of government policy priorities and how and to what extent does it influence their research? How does this vary across different disciplines?
89. How do the changing needs of policy-makers and limited timeframes of government policy impact the development and funding of long-term research and research infrastructure.
90. In thinking about how the school curriculum can be developed to keep pace with advancing science and technology, how can the scientific community work with policymakers in setting priorities in science education in schools?
91. In thinking about how young people can be engaged with science and technology in further education and training, how can the scientific community work with policymakers in setting priorities in education and training?
92. Given the importance of keeping pace with scientific and technological advances for maintaining global competitiveness, how can the scientific community work with policymakers to sustain and develop the scientific literacy of the workforce?
93. Given the importance of keeping pace with scientific and technological advances for informed citizenship, how can the scientific community work with policymakers and national and local organisations to sustain and develop the scientific literacy of the nation?
94. How can the scientific community work with teachers and policymakers to ensure that future generations are engaged with science and technology?
95. How can the scientific community work with teachers and policymakers to ensure that future generations are empowered by science and technology?
96. How can the scientific and science-education community work with policymakers to put in place strategic planning at all levels of education so as to anticipate the future needs of the nation?
97. How can policymakers and other stakeholders work together to set priorities in scientific and social-scientific research in such a way as to anticipate the future needs of the nation?
98. How can policymakers, higher education institutions and other stakeholders work together to sustain and grow the science base?
99. What role should scientific evidence play in policymaking?
100. Should the extent to which scientific evidence is used in policymaking change according to the nature and the context of the policy decision being made?
101. What kinds of evidence would policymakers need to understand in order to ensure good governance?
102. What kinds of evidence would policymakers need in order to make informed decisions?
103. Are different kinds of evidence required/sought to support strategic planning and policymaking at different levels of governance (e.g. national, regional, local, city, borough, county, town)?
104. How can policymakers work with scientific experts to embed the use of evidence into the policymaking process?
105. What part should knowledge of science and its modes of operation play in the continuing professional development of policymakers and policy officials at all levels of government?
106. How can scientific experts work with policymakers to ensure that we continue to capitalise on scientific and technological advances in a sustainable way so as to promote economic prosperity and improve quality of life and well-being?
107. How can policymakers work with industry, business and other interest groups to enhance and enrich science education in schools whilst ensuring the delivery of a balanced science curriculum?
108. Can we think of a formal platform where the scientists and policy makers sit together and discuss science policy issues periodically? On significant scientific issues, while the scientists sit together in conferences and seminars and discuss, the politicians gather in G8s and G20s.
109. Should science policies be made people-centric by involving the public in pre-policy discussions (e.g. crowdsourcing)? If so, what types of platforms would be most effective for these discussions?
110. Given the concept that we are increasingly living in a "global village" and seek to commercialise innovation in foreign markets, how important is it for UK to achieve standardisation of patent policies and laws across international borders? And how feasible would this be?
111. The House of Commons Select Committee on Science and Technology was established in 1992. What have the effects of this Select Committee been on the development of science and technology in the UK? How are those effects measured?
112. Think tanks tend to set or expand the limits of respectable debate. There are a lot more think tanks today (over 5,000 worldwide at last count) than there were in the 1970s. What are the underlying reasons for this growth pattern? More importantly, how can a feedback mechanism or a framework of metrics be developed in order to help measure the relevance of think tanks' contributions in shaping science policy decisions?
113. How exactly does the precautionary principle relate to the “sound science” notion in regulatory decisionmaking, i.e., are they really in opposition?
114. What are the different ways that the precautionary principle is effected in regulation, e.g., how is it setting different kinds of evidentiary burdens in law and regulatory policy?
115. What are the conditions under which an expert scientific body is trusted or not trusted by society?
116. How are non-governmental organizations participating in the construction of the scientific basis of policy? Corporate actors?
117. How are experts actually chosen for high-level blue-ribbon committees on technical matters? How should they be chosen?
118. How does expertise operate in the emerging global administrative system for health, food, environment, etc? What does / should ‘representation’ and participation in global expert agencies look like for developing countries?
119. How and where is ‘extended peer review’ or ‘post-normal science’ being put into operation? How is it doing?
120. What does democratizing science mean outside of “public participation”? What are potential other mechanisms to make science advice more accountable to and responsive to societal values?
121. How are definitions of what counts as ‘science’ in the regulatory arena changing?
122. Who sets the precise agendas for science funding? Who should do so, given that both societal values and technical knowledge are relevant?
123. Compare and contrast how science advisory bodies operate in US, EU, Asia and the rest of the world.
124. What constitutes a valid form of evidence acceptable to support evidence-based policy?
125. What factors enable particular metrics of risk assessment to be recognised as objective and scientific?
126. What evidence is there that policy has, on occasion, been enacted in the absence of evidence? Has this resulted in negative consequences?
127. What evidence is there of policy outstripping the capacity of science to deliver supporting knowledge?
128. In what circumstances and how can lay knowledge make important contributions to policy development?
129. Is it still feasible to consider the introduction of a "public understanding of science" agenda? If so, what forms might it take?
130. Has there been a loss of public confidence in scientific inputs into policy? If so, what have been the causes of this, and what have been the key moments in this process? And, if there can be shown to be a loss of confidence, is it reversible, and if so, how?
131. What differences may arise in the science-policy arena following the abandonment of a simplified linear model of "science into policy"?
132. What factors enable a claim about a particular problem that is labelled by its protagonist as objective and “science-based” to be recognised as such in the political sphere?
133. What transformations are needed in the modes of communication of scientific knowledge to facilitate its reception by different actors and agents, and in different sectors?
134. What mechanisms can researchers deploy to ensure that their work is policy relevant, without being policy driven?
135. In what circumstances would it be advantageous to slow down the translation of science into policy and/or technology, and what might the consequences of this be?
136. How are the scientific agendas of the research councils developed, and what role do the wider scientific community, the policy community and industry play in this process? What are the advantages and disadvantages for the science-policy arena of different models for the setting of research agendas?
137. What are the roles boundary institutions are perceived to play in mediating between science and policy, and are they perceived differently in the two communities?
138. What roles do space and place play in the drawing of conclusions from empirical scientific research, and what might be the implications of this for the use of science in policy? If these factors are important, are they recognised as such by actors at the science/policy interface?
139. If current arguments in favour of incorporating human behaviour in global change models are accepted, what changes might be necessary in the way in which model outputs are used to inform policy?
140. Who/which actors at the science/policy interface should be granted responsibility for judging whether uncertainties in relevant bodies of scientific knowledge are of policy relevance or not?
141. Arguments in support of inter-disciplinary approaches to policy problems are expected to move the consideration of social science issues "upstream"; what are the implications of this for science and policy?
142. What methods are likely to be most successful for exposing the range of sources of uncertainty in scientific knowledge, and how are these different sources likely to be accommodated in the policy process?
143. What should be the basis for political decision-making when scientific data and evidence is unable to provide a clear answer to a complex or “wicked” problem?
144. How can the scientist best communicate the degree of certainty (or uncertainty) of scientific claims to policy makers?
145. How can policy makers best communicate the social benefits (or costs) of acting on a scientific claim if it is right (or wrong
146. Can the scientist assess the degree of certainty (or uncertainty) of a scientific claim without reference to its uses in policy making?
147. Policy-making in government is a complex process in which science is but one of a large number of influences. How can we best build an objective, auditable and visually demonstrable understanding of this process?
148. How can we then best improve the likelihood that scientific advice and evidence is appropriately considered in policy?
149. What is the evidence for the evidence-base being used as a key factor in decision-making – review?
150. In the current coalition Government how might the balance between statutory and Third Sector advice change, and what are the likely implications?
151. How do we break out of the cycle where policy questions require significant research before answer?
152. What is the mechanism for linking policy and science customers within Government, and between Government and external science advisers?
153. Have recent experiments in public dialogue with science and technology had a demonstrable impact on the governance of science?
154. Do politicians and civil servants see experts as playing a purely advisory role or a 'challenge' role?
155. What are the relative advantages of narrow expert groups and broad-based multidisciplinary groups in shaping and offering advice?
156. What is the role of open access to scientific publications and data in strengthening the public credibility of science?
157. How are blogs changing public engagement with policy-relevant science?
158. How do we formally express the limits and uncertainties of science in understanding and tackling policy problems?
159. How should scientists talk about uncertainties that can't be characterised scientifically?
160. How do we construct a normative theory of expertise that allows for the political complexities of public science issues?
161. How should policies strike a balance between collaboration and competition in global networks of science?
162. How should the tensions between universal knowledge/local knowledge, standardisation/localisation etc. be managed between countries?
163. What is the role of science in defining, meeting and governing global challenges?
164. Is the practice of science universal? Do, and if so how do, different countries produce different sorts of science?
165. Is democracy a necessary condition for science? Can science flourish under non-democratic regimes?
166. What is the role of National Academies at the start of the 21st century? How has this changed? What models of expertise and authority do they embody?
167. What role can science play in international diplomacy, network building and soft power?)
168. What has been the contribution of Science and Technology Studies/Science Policy Studies to improved governance?
169. Are sustainability and innovation in conflict?
170. Does the governance of research mean ‘slowing down’ innovation?
171. How should priorities be set for scientific input to policy? Should these be driven by policy makers’ priorities? Or by a broader assessment of the relative social importance of a given issue?
172. How can scientific advice be maintained over time on long term issues (eg climate change), or those that are socially important but have little political gain, in the face of changing political priorities?
173. How might priorities be better assessed? Should this be done within a given policy area (eg environment, health, security etc)? Who is best placed to judge and define these priorities?
174. How far should scientific advice/input be driven by an ethical standpoint? Should this be articulated? Should this restrict advice in particular areas?
175. What are the “understanding gaps” that exist between the scientific and policy (or political or public) communities across a range of disciplines? Representative sub-questions: What is the perception and general level of understanding among scientists of policy processes across different disciplines? What is the perception and general level of understanding among the policy (public, political, media) community about scientific processes across different disciplines? How do these understanding gaps vary between different cultural or national contexts, and or at different levels of government (municipal through federal) within nations?
176. How effective have national and international Scientific Panels been at inducing evidence-based policy decisions? Representative sub-questions: Are panel processes more effective when requested or convened by governments, by civil society organizations (NGOs) or by scientific bodies (Academies)? What processes have been most effective at ensuring the relevance/saliency of panel outputs to the relevant policy, political, media and civil society communities?
177. What new training programs could make the next generation of scientists and policymakers more effective at interfacing scientific knowledge generation with 21st century public policy challenges?
178. What are the various ‘roles’ scientists can play when attempting to inject knowledge into policymaking processes? (e.g. a range from neutral technical advisor through to public advocate for particular policies) What are the implications – particularly across different disciplinary (e.g. medicine versus ecology) and national/cultural contexts – of these roles for (a) the efficacy of the scientist in influencing policy and (b) for the career of the scientist?
179. What are the various forms of “boundary” organization and individual that mediate/translate between the scientific and policy/political/media/public communities? What [structures and processes] (skill sets) make these [organizations] (individuals) most effective at influencing policy/political outcomes? What training programs could be developed to support/enhance such boundary activities? In what contexts are such boundary activities not required?
180. Defining ways to measure historical returns to public sector R&D, particularly R&D associated with environmental and security benefits. There are too few studies focused on the specific (rather than aggregate) benefits of government-funded R&D (e.g., NRC 2001 report "Energy Research at DOE: Was it worth it?). Novel ways of measuring benefits and new case studies quantifying them could help governments all over the world provide a more stable support for innovation.
181. Analyzing what conditions or actors have resulted in large science policy changes. For example, support for hydrogen energy research in the United States surged starting in 2002 and was significantly diminished by 2010. What information are policy makers relying on? What coalitions were formed or dissolved? What can we learn to design programs with an "exit strategy"? The question of designing R&D policy that is stable but flexible and adaptive has not been sufficiently answered.
182. Investigating how government programs should draft funding opportunity announcements for R&D grants or partnerships. It is not trivial to ensure that government R&D grants are the grants of today and not the grants of yesterday. Gathering information about the result of previous grants, and incorporating private sector expertise as well as academic expertise are crucial. Are there any good models for this? Japan has an interesting roadmapping process to support industrial research.
183. Assessing the impact of expert assessments on specific science policies. For example, the United States Congress called in 2008 for an assessment of the information on climate change science and to make recommendations about what steps could be adopted. This request resulted in the America's Climate Choices report. Essentially it is a U.S. version of the IPCC with some policy recommendations. The report did not receive a lot of press or unleash a lot of policy activity. Was this a useful addition to the IPCC or a good response to the skepticism that the IPCC report unleashed? What are the characteristics of topic, political and social circumstances, panel design, and information dissemination that increase the chances that expert panels will result in policy action?
184. Another question that has come up that is very difficult to tackle is whether science policy researcher can do anything to help governments prioritize between supporting innovation in different areas (e.g., health, defense, environment, climate change) to different extents.
185. How can we ensure that the research community have appropriate knowledge/training in the policy process/appropriate policy language in order to be able to influence it?
186. How should we communicate scientific topics to a policy audience?
187. What mechanisms do we use to ensure the quality of research used to inform policy? i.e) do we only use peer-reviewed research - (if so, who should assemble the evidence?) or do we use a specific expert or group of experts?
188. Researchers are often very adept at talking about solutions (their research), and not problems (the challenges their research/solution can address), how can we improve this?
189. Related to this - how do we improve the level of understanding of the link between fundamental science and benefit to society? E.g. how does research into protein engineering benefit patients in the NHS?
190. What can the scientific community do to make themselves seen as an available, valuable and user-friendly source of evidence?
191. How is [should] scientific advice [be] weighed against other forms of evidence, and other considerations, in arriving at policy decisions?
192. How should processes of ‘evidence informed policy’ respond to different knowledge and policy contexts, for example as characterised by levels of scientific uncertainty and issue contestation?
193. How can we design better processes of dialogue between policy makers and researchers to identify research questions, such that the answers will be of real value to policy makers?
194. How should we characterise excellence in research aimed at informing policy decisions?
195. How can scientific assessments for policy be made less captive to in-group and out-group effects?
196. What empirical evidence is there that public scrutiny of expert evidence leads to more enduring and legitimate policy measures?
197. What guidelines may be offered for matching different methods of scientific consensus-making to different types of policy arenas/questions?
198. How can public expectations of science as truth-machine and scientists as truth-tellers be modified in line with what experts know is the provisionality of knowledge?
199. In what ways can humanities traditions and scholars contribute to expert science panels for policy?
200. How do the context, nature and framing of evidence influence policy-making?
201. What is the impact of scientific uncertainty and disagreement on policy-making?
202. How can scientific uncertainty and disagreement best be expressed as part of policy discourse?
203. What factors within the policy process determine the weight policy makers give to scientific advice?
204. What factors within the policy process determine whether and how much scientific advice is sought on a problem? Is there a fundamental problem with the different timescales of scientific and policy processes?
205. How does the existence of in-house scientific expertise (for instance experts in the Scientific Civil Service) affect the quality of scientific advice, and how it is handled? How do other countries manage this issue?
206. How much scientific advice on policy issues is provided by: a) academics; b) consultancies; c) in house Govt scientists; d) other. What is the impact of the choice of source?
207. How much of the process of provision of expert advice should be open to public scrutiny? And at which stage?
208. How well do policy makers understand scientific advice?
209. How do we guard against “policy based evidence-making?
210. When and under what conditions is policy advice most likely to have effect?
211. When and under what conditions is evidence to inform policy considered 'sound'?
212. How do different approaches to framing policy problems affect the quality of the advisory process?
213. What models exist for the inclusion of public views and values in technically complex advisory processes?
214. What are the consequences for the relationship between science and policy of differences between national political cultures?)
215. What criteria can be used to assess the social and technical robustness of scientific advice?
216. In exploring the connections between 'science' and 'policy', how do we delineate these categories? Are boundaries more difficult to maintain in some cases than in others?
217. How can established mechanisms for priority setting in science respond to potential future needs of policy?
218. When grappling with complex issues in democratic societies, does the promotion of evidence-based policy inevitably slide towards technocracy?
219. Which kinds of risk assessment policies (&/or benefit assessment policies) have been adopted by particular institutions such as policy-makers, individual expert scientific advisors and advisory bodies?
220. In which institutional locations are risk (&/or benefit) assessment policies being decided? By which process are they legitimated or accountable?
221. How have those risk &/or benefit assessment policies evolved? And how have the locations at which they are set, and the processes by which they have been set, changed?
222. How and where are decisions taken on the issue of how much evidence, and which kinds of evidence, are deemed sufficient to recommend for example approval, rejection or restriction?
223. Is the advice of scientific experts to policy-makers expected to inform decisions or dictate them? Do the expectations of the advisors and of the policy-makers coincide?)
224. To what extent is the evidence on which expert advisors deliberate in the public domain? Who decides, and how, what is disclosed and what is concealed?
225. To what extent do policy-makers and/or their expert advisers reveal symmetries or asymmetries in their concern with identifying possible false positives and false negatives? Are they symmetrically sensitive to those two possibilities or more focussed on one side than the other?
226. To what extent do scientific advisors acknowledge scientific uncertainties? Are the uncertainties comprehensively acknowledged or selectively acknowledged? If selectively, what influences those selections?
227. What are the processes by which policy-makers decide which are the specific questions that they ask of their expert advisors? By which processes are those decisions accountable?
228. How important is the conservation of biological diversity, at the national level, to economic development?
229. Which ecosystem services depend on biological diversity, and how can that dependence be quantified?
230. Do protected areas negatively impact the livelihoods of people living in and around the area?
231. Under what circumstances do protected areas contribute to national economic development?
232. How can large-scale infrastructure projects under present conditions of national development be designed to minimize impact on natural areas?
233. Under what conditions should PES (Payments for Ecosystem Services) initiatives be managed by centralized national institutions, and under what conditions should management be decentralized to municipalities, local communities, etc.?
234. When do CSR (Corporate Social Responsibility) policies and practices positively contribute to the conservation of biodiversity, and when do they simply minimize negative impact?
235. Can scientific assessments for policy ever be neutral on the policy options?
236. What is the correlation between the length of a science policy document and its impact on a policy decision?
237. Does having a chief scientific adviser change how science is viewed and used by ministers?
238. Are there aspects of policy for science, which are beyond the scope of industrial policy?
239. Is spending on science an appropriate surrogate measure for commitment to science?
